# Quantum engineered Kondo lattices

**DOI:** 10.1038/s41467-019-13446-1

**Published:** 2019-12-06

**Authors:** Jeremy Figgins, Laila S. Mattos, Warren Mar, Yi-Ting Chen, Hari C. Manoharan, Dirk K. Morr

**Affiliations:** 1 Department of Physics, University of Illinois at Chicago, Chicago, IL 60607 USA; 2 Stanford Institute for Materials and Energy Sciences, SLAC National Accelerator Laboratory, Menlo Park, CA 94025 USA; 3 Department of Physics, Stanford University, Stanford, CA 94305 USA; 4 Department of Electrical Engineering, Stanford University, Stanford, CA 94305 USA; 5 Department of Applied Physics, Stanford University, Stanford, CA 94305 USA

**Keywords:** Electronic properties and materials, Magnetic properties and materials

## Abstract

Atomic manipulation techniques have provided a bottom-up approach to investigating the unconventional properties and complex phases of strongly correlated electron materials. By engineering artificial systems containing tens to thousands of atoms with tailored electronic or magnetic properties, it has become possible to explore how quantum many-body effects emerge as the size of a system is increased from the nanoscale to the mesoscale. Here we investigate both theoretically and experimentally the quantum engineering of nanoscale Kondo lattices – Kondo droplets – exemplifying nanoscopic replicas of heavy-fermion materials. We demonstrate that by changing a droplet’s real-space geometry, we can not only create coherently coupled Kondo droplets whose properties asymptotically approach those of a quantum-coherent Kondo lattice, but also markedly increase or decrease the droplet’s Kondo temperature. Furthermore we report on the discovery of a new quantum phenomenon – the Kondo echo – a signature of droplets containing Kondo holes functioning as direct probes of spatially extended, quantum-coherent Kondo cloud correlations.

## Abstract (web summary)

The mechanism underlying screening of a Kondo lattice of magnetic moments by conduction electrons is an important unsolved problem. Figgins et al. use STM techniques to assemble nanoscale Kondo droplets that extrapolate between a single moment and the Kondo lattice, providing a platform for further study.

## Introduction

Quantum engineering of finite-size artificial adatom lattices^1–7^ on metallic surfaces has provided a unique approach to explore how variations in the lattice shape or structure affect emergent electronic properties. This has given rise to the creation of Dirac cones in molecular graphene^5^ and artificial Lieb lattices^6^, as well as the discovery of many quantum phenomena, such as quantum mirages in Kondo corrals^1,2^ or topological superconductivity^7^. Artificial lattices also open a new road to studying the emergence of strong correlation effects^8–12^ through the use of magnetic adatoms, and the ensuing Kondo effect^13^. Such Kondo lattices^4^, which when rendered coherent, are believed to contain all salient features of heavy-fermion materials. They should permit the exploration of how many-body phenomena emerge at the nanoscale, evolve across the mesoscale, and result in the many complex properties of macroscopic systems^14^. At the same time, atomic manipulation techniques enable the controlled implementation of defects or vacancies, opening up a new field for studying the interplay between disorder and strong correlation effects. These possibilities might hold the key to greatly advancing our understanding of the many unconventional properties and complex phases of strongly correlated electron materials at the macroscale^9,10^.

In the following, we demonstrate theoretically and experimentally how the properties of Kondo droplets are affected by the droplet’s real-space geometry and evolve with increasing droplet size. In particular, we show that by changing a droplet’s real-space geometry, we can (a) quantum engineer coherently coupled Kondo droplets whose properties asymptotically approach those of a quantum-coherent Kondo lattice, and (b) markedly increase or decrease the droplet’s Kondo temperature. As these coherent Kondo droplets can be created with fewer than 50 adatoms, our results open an arena for the exploration of heavy-fermion physics at the nanoscale. Furthermore, we demonstrate that a Kondo echo emerges at Kondo hole sites in a Kondo droplet as a characteristic signature of spatially extended, quantum-coherent Kondo cloud correlations.

## Results

### Large-*N* theory for Kondo droplets

**Fig. 1:**
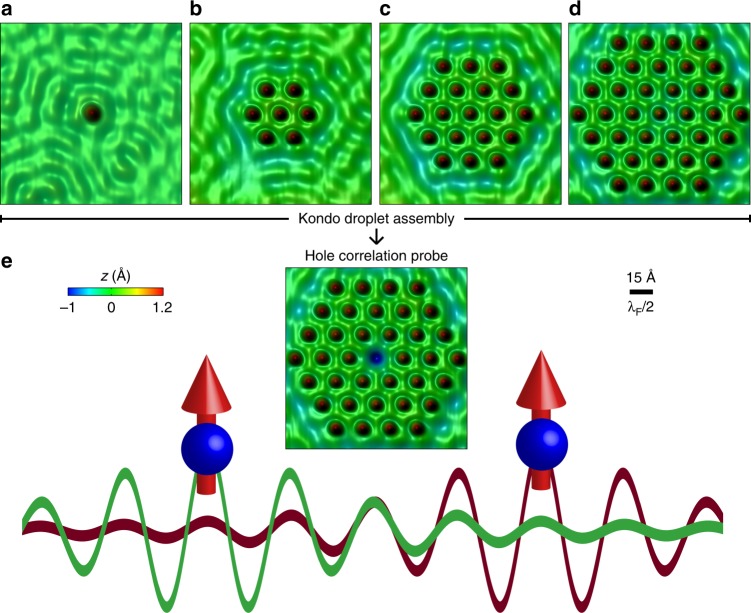
Assembly of Kondo droplets using atomic manipulation. Starting from a single Co adatom **a** consecutive construction of a Kondo droplet with 1 (**b**), 2 (**c**), and 3 (**d**) rings of Co adatoms. In **e** a single atom is removed to create a Co vacancy (Kondo hole) in the droplet center, used as a Kondo cloud correlation probe for the entire droplet. Here, the Co adatom distance is $\Delta {r}_{{\rm{2}}}=4\sqrt{3}{a}_{0}\approx 17.7$ Å, with ${a}_{0}=2.55$ Å being the Cu(111) surface nearest-neighbor spacing at $T=4.2$ K. Colorbar indexes measured tip height $z$. Right scale bar indicates the length scale of one-half Fermi wavelength of the itinerant 2D electron gas. Schematic: Quantum interference of electronic wave functions (red and green lines) participating in the Kondo screening of magnetic Co adatoms (filled blue circles with red arrows) on nearby sites can either enhance or suppress Kondo screening. In the case of constructive interference, a macroscopic Kondo cloud pervades the entire droplet and is detected at the Kondo hole site as a Kondo echo.

**Fig. 2:**
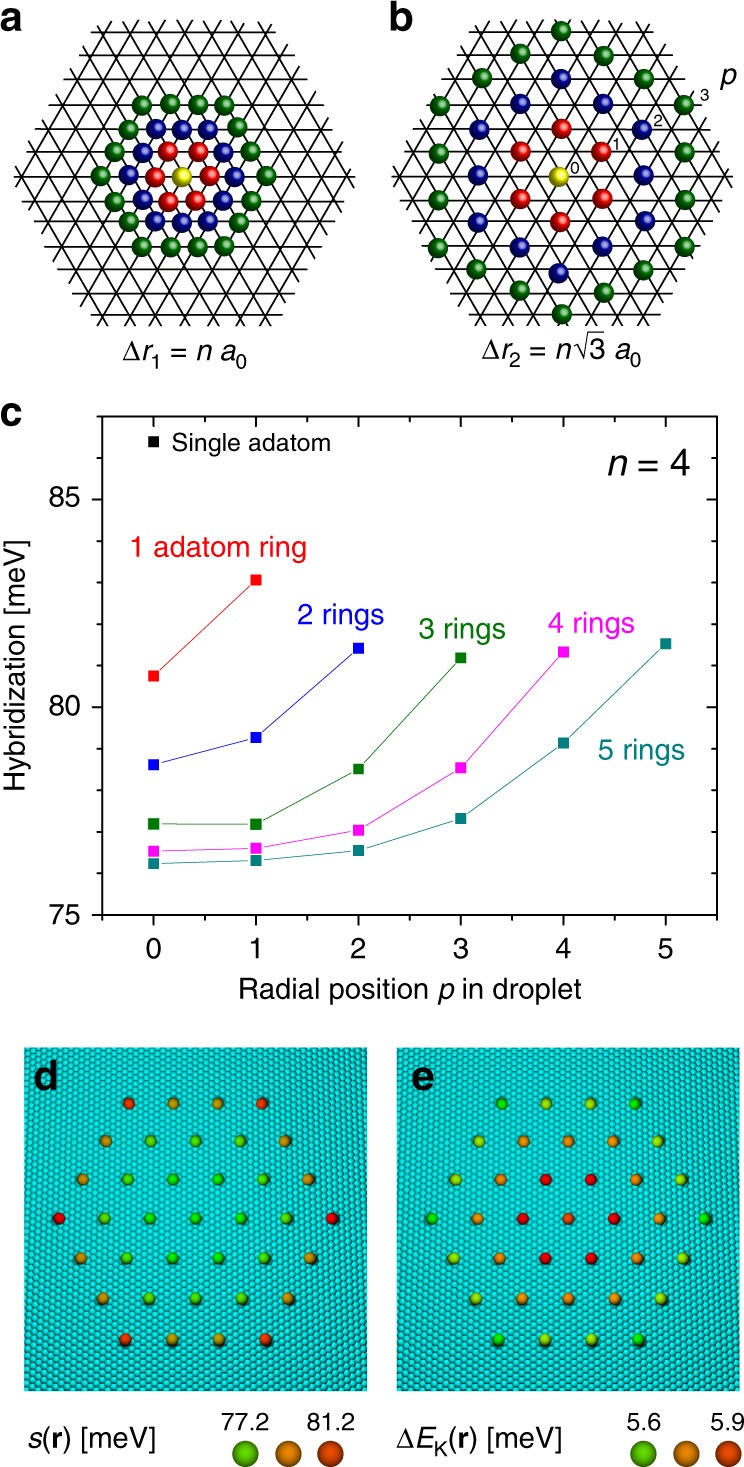
Spatially dependent Kondo screening within a droplet. Spatial structure of Kondo droplets consisting of three rings of magnetic adatoms with distance **a** $\Delta {r}_{{\rm{1}}}=n{a}_{0}$ and **b** $\Delta {r}_{{\rm{2}}}=\sqrt{3}n{a}_{0}$, with $n$ integer, here shown for $n=1$. For clarity, the rings are shown using different adatom colors. **c** Hybridization $s({\bf{r}})$ for different sites in the Kondo droplet shown in **b** with $\Delta {r}_{{\rm{2}}}=4\sqrt{3}{a}_{0}$ with increasing number of rings of magnetic adatoms. Spatial structure of **d** $s({\bf{r}})$ and **e** $\Delta {E}_{{\rm{K}}}({\bf{r}})$ for a Kondo droplet with three rings of adatoms and $\Delta {r}_{{\rm{2}}}=4\sqrt{3}{a}_{0}$.

To study the emergence of correlation effects in Kondo droplets, we use atomic manipulation to arrange magnetic Co adatoms on metallic Cu(111) surfaces^1^ in the form of highly ordered, hexagonal Kondo droplets (Fig. 1a–d). After benchmarking the intact lattices, we study defects in the form of Kondo holes created through missing Co adatoms (Fig. 1e). Cu(111) surfaces are uniquely suited to investigate the emergence of many-body effects in Kondo droplets, as Kondo screening arises from the coupling to a surface band of two-dimensional electrons^1^—in which correlation effects are expected to be spatially longer ranged—rather than to three-dimensional bulk bands. Such Kondo droplets are described by the Kondo Hamiltonian^8,13,15–20^

1 $${\mathcal{H}}=\sum \limits_{{\bf{r}},{\bf{r}}^{\prime} ,\sigma }\left(-{t}_{{\bf{r}}{\bf{r}}^{\prime} }-\mu {\delta }_{{\bf{r}}{\bf{r}}^{\prime} }\right){c}_{{\bf{r}},\sigma }^{\dagger }{c}_{{\bf{r}}^{\prime} ,\sigma }+J {\sum\limits _{{\bf{r}}}}^{\prime} {{\bf{S}}}_{{\bf{r}}}^{{\rm{K}}}\cdot {{\bf{s}}}_{{\bf{r}}}^{{\rm{c}}},$$

where $${c}_{{\bf{r}},\sigma }^{\dagger }$$$$({c}_{{\bf{r}},\sigma })$$ creates (annihilates) a conduction electron with spin $$\sigma$$ at site $${\bf{r}}$$ on the Cu surface. Here, $${t}_{{\bf{r}}{\bf{r}}^{\prime} }=0.924$$ eV is the fermionic hopping element between nearest-neighbor sites in the triangular Cu(111) surface lattice, and $$\mu =-5.13$$ eV is its chemical potential, yielding a Fermi wavelength of $${\lambda }_{{\rm{F}}}\approx 11.5{a}_{0}$$, where $${a}_{0}$$ is the Cu lattice constant^2,5^. Moreover, $$J\, > \, 0$$ is the Kondo coupling, and $${{\bf{S}}}_{{\bf{r}}}^{{\rm{K}}}$$ and $${{\bf{s}}}_{{\bf{r}}}^{{\rm{c}}}$$ are the spin operators of the magnetic Co adatom and the conduction electron at site $${\bf{r}}$$, respectively. The primed sum runs over the locations of the Co adatoms only. We note that the lattice structure of the Cu(111) surface allows for the creation of two natural types of hexagonal Kondo droplets (which are rotated by $$3{0}^{\circ }$$ with respect to each other) with adatom distances of $$\Delta {r}_{{\rm{1}}}=n{a}_{0}$$ (see Fig. 2a) and $$\Delta {r}_{{\rm{2}}}=n\sqrt{3}{a}_{0}$$ (see Fig. 2b) with $$n$$ being an integer.

To describe the Kondo screening of the Co adatoms by Cu(111) surface electrons, we employ a large-*N* expansion^15–17,20–23^, which has previously been used to successfully explain^24,25^ the asymmetric form of the Kondo resonance in the differential conductance, ${\mathrm{d}}I/{\mathrm{d}}V$, measured via scanning tunneling spectroscopy (STS). Here, ${{\bf{S}}}_{{\bf{r}}}^{{\rm{K}}}$ is generalized to $SU(N)$ and represented via Abrikosov pseudofermion operators ${f}_{{\bf{r}},\alpha }^{\dagger },{f}_{{\bf{r}},\alpha }$, which obey the constraint ${\sum }_{\alpha =1..N}{f}_{{\bf{r}},\alpha }^{\dagger }{f}_{{\bf{r}},\alpha }=1$ with $N=2S+1$ being the spin degeneracy of the magnetic adatom. This constraint is enforced at every site of the Kondo droplet by means of a Lagrange multiplier ${\varepsilon }_{{\rm{f}}}({\bf{r}})$, while the exchange interaction in Eq. (1) is decoupled via a hybridization field, $s({\bf{r}})$. The hybridization represents the hopping amplitude between the conduction electron states and the pseudofermion $f$-electron states and reflects the strength of the Kondo screening. In particular, for a single Kondo impurity, the onset temperature for Kondo screening, the Kondo temperature ${T}_{{\rm{K}}}$, is related to the width of the Kondo resonance, $\Delta {E}_{{\rm{K}}}$, in the differential conductance (see below) via ${k}_{\rm{B}}{T}_{{\rm{K}}}=\Delta {E}_{{\rm{K}}}$, with the latter scaling as $\Delta {E}_{{\rm{K}}} \sim {s}^{2}$ (see ref. ^16^). For fixed $J$, ${\varepsilon }_{{\rm{f}}}({\bf{r}})$ and $s({\bf{r}})$ are obtained on the saddle point level by minimizing the effective action^21^ [for details see Supplementary Note 1]. We note that the Ruderman–Kittel–Kasuya–Yosida (RKKY) interaction between magnetic moments^8,16,19^ for the two-dimensional (2D) Cu(111) surface band decays rapidly with increasing distance^26^, and is significantly smaller than ${k}_{\rm{B}}{T}_{{\rm{K}}}$ for the relevant inter-adatom distances considered below, in agreement with experimental findings^27^, such that this interaction can be neglected. Finally, to reproduce the lineshape and width of the Kondo resonance measured experimentally^1^ for a single Co adatom on a Cu(111) surface, we employ $J=3.82$ eV and $N=4$ (see below and ref. ^24^).

### Evolution of Kondo screening with increasing droplet size

To investigate the evolution of Kondo screening from a single Kondo impurity to the Kondo lattice, we begin by considering how the droplet’s physical properties change with increasing number of rings of Co adatoms with adatom distance $\Delta {r}_{{\rm{2}}}=4\sqrt{3}{a}_{0}$ (see Fig. 2b). To this end, we present in Fig. 2c the self-consistently computed hybridization, $s({\bf{r}})$, for different adatom sites in the Kondo droplet (as denoted in Fig. 2b) and increasing number of adatom rings. A key physical fact that emerges is that the hybridization in the center of the droplet—denoted as site $p=0$—decreases with increasing number of rings, with the decreasing rate of change between consecutive rings indicating that it approaches an asymptotic value. As the hybridization for a single Kondo adatom is in general larger than that for a Kondo lattice (assuming the same set of parameters) – essentially since the conduction electrons take part in the Kondo screening of multiple magnetic adatoms – this decrease of $s$ with increasing droplet size reflects the crossover in the droplet’s properties from those of a single Kondo impurity to those of a Kondo lattice. Moreover, as one moves from the droplet’s center to its edge, the hybridization increases, indicating that the properties of the Kondo adatoms along the edge lie between those of the bulk (as exemplified by the droplet’s center) and those of a single Kondo impurity. In Fig. 2d, e, we present a spatial plot of $s({\bf{r}})$ and of the width of the Kondo resonance, $\Delta {E}_{{\rm{K}}}({\bf{r}})$, at the site of the Co adatoms, respectively, for a Kondo droplet with three rings of magnetic adatoms [$\Delta {E}_{{\rm{K}}}({\bf{r}})$ is extracted by fitting the Fano formula^28^ to the theoretical ${\mathrm{d}}I/{\mathrm{d}}V$ lineshape, as described in Supplementary Note 2]. It is intriguing that these two properties are anti-correlated: while $s({\bf{r}})$ is smaller in the center of the droplet than at the edge (for the reasons discussed above), $\Delta {E}_{{\rm{K}}}({\bf{r}})$ is larger in the center. Indeed, the larger value of $\Delta {E}_{{\rm{K}}}({\bf{r}})$ in the center, reflecting the coherent coupling of individual Kondo resonances, is another signature of the crossover to a Kondo lattice: with increasing number of rings, the suppression of the density of states in the Kondo resonance becomes larger and more extended in energy as it evolves into the hybridization gap^29^. The anti-correlated spatial structure of $s({\bf{r}})$ and $\Delta {E}_{{\rm{K}}}({\bf{r}})$ shown in Fig. 2d, e, therefore, directly reflect the different physical properties of a Kondo impurity and a Kondo lattice. These results thus imply that it is possible to study the crossover from a single Kondo impurity to the Kondo lattices not only with increasing droplet size, but also within the same droplet exhibiting bulk properties in its center and properties more similar to isolated impurities along its edges.

### Quantum-coherent and incoherent Kondo droplets

**Fig. 3:**
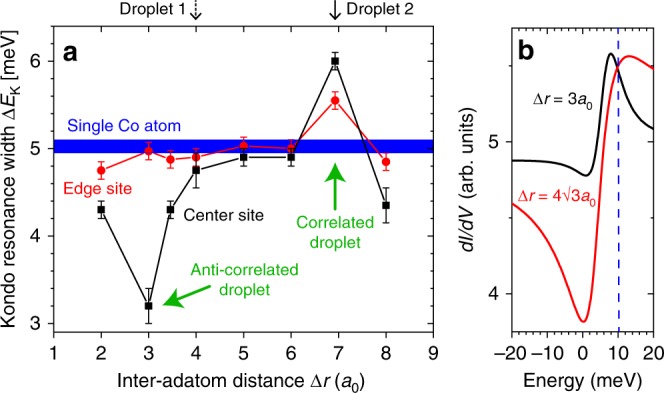
Engineering the strength of Kondo screening. **a** Dependence of $\Delta {E}_{{\rm{K}}}({\bf{r}})$ (which is a measure for the strength of the Kondo screening) at the center (black line/symbols) and edge sites (red line/symbols) of a Kondo droplet with three rings on the inter-adatom distance $\Delta r$. $\Delta {E}_{{\rm{K}}}({\bf{r}})$ has been extracted by fitting the Fano formula [Eq. (12) in Supplementary Note 2] to the theoretically computed ${\mathrm{d}}I/{\mathrm{d}}V$. Vertical bars indicate the uncertainty in $\Delta {E}_{{\rm{K}}}({\bf{r}})$. The width of the blue line indicates the uncertainty in $\Delta {E}_{{\rm{K}}}({\bf{r}})$ for an isolated Co adatom. **b** ${\mathrm{d}}I/{\mathrm{d}}V$ at the center site of a three-ring Kondo droplet for $\Delta r=4\sqrt{3}{a}_{0}$ (red curve, maximally correlated droplet) and $\Delta r=3{a}_{0}$ (black curve, anti-correlated droplet). These two lattices correspond to green arrows in **a** contrasting the droplet behavior for constructive (correlated droplet) and destructive (anti-correlated droplet) quantum interference. At other lattice spacings (see **a**), the droplet is uncorrelated and Kondo screening occurs essentially independently at each adatom site. The vertical black arrows above **a** indicate the experimentally realized droplets, which contrast uncorrelated (dashed arrow, droplet 1) and correlated (solid arrow, droplet 2) Kondo screening.

The hybridization as well as the width of the Kondo resonance, $\Delta {E}_{{\rm{K}}}$, can be controlled in a non-monotonic fashion, by varying the distance between the magnetic adatoms, $\Delta r$. To demonstrate this, we present in Fig. 3a the width of the Kondo resonance, $\Delta {E}_{{\rm{K}}}({\bf{r}})$ (see Supplementary Note 2), at the center and edge sites (the latter denoted as site $p=3$ in Fig. 2b) of a Kondo droplet with three rings for increasing inter-adatom distance. Not only is the dependence of $\Delta {E}_{{\rm{K}}}$ on $\Delta r$ non-monotonic, but $\Delta {E}_{{\rm{K}}}$ can also be significantly enhanced or suppressed from that of a single Kondo impurity (thick blue line), with the largest enhancement and suppression at the center site occurring for $\Delta r=4\sqrt{3}{a}_{0}$ and $\Delta r=3{a}_{0}$, respectively. The large difference in $\Delta {E}_{{\rm{K}}}$ for these two Kondo droplets is clearly revealed in the ${\rm{d}}I/{\rm{d}}V$ lineshape at the center sites, shown in Fig. 3b. This non-monotonic behavior of $\Delta {E}_{{\rm{K}}}$ arises from the constructive or destructive quantum interference between the Kondo screening clouds associated with each individual magnetic adatom (Fig. 1e and Supplementary Note 3). We note that with increasing droplet size, the width of the Kondo resonance increases and evolves into the hybridization gap of the coherent Kondo lattice^29^. As such, the increased $\Delta {E}_{{\rm{K}}}$ for $\Delta r=4\sqrt{3}{a}_{0}$ is strong evidence for the coherent and constructive coupling of the Kondo screening clouds associated with the individual magnetic adatoms, and hence the creation of a coherent Kondo droplet hosting extended Kondo cloud correlations. On the other hand, for $\Delta r=3{a}_{0}$ the Kondo resonance at the center of the droplet is strongly suppressed, indicating the destructive interference of the Kondo screening clouds. This demonstrates that the creation of a coherent, nanoscale Kondo lattice can be controlled through manipulation of the droplet’s geometry and the ensuing quantum interference processes.

### Experimentally engineered Kondo droplets

**Fig. 4:**
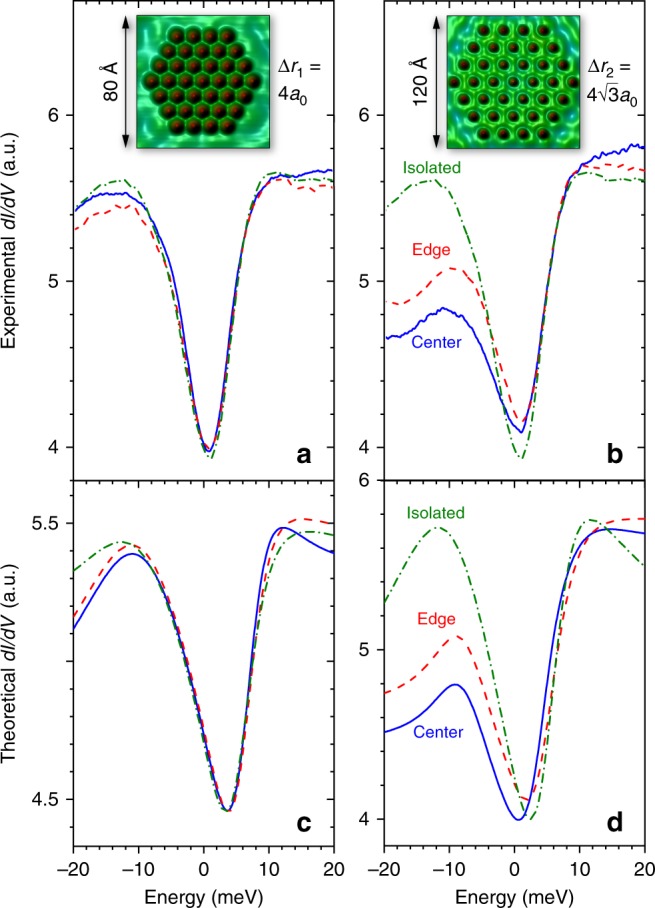
Observation of enhanced Kondo screening. **a** Experimental ${\mathrm{d}}I/{\mathrm{d}}V$ spectra measured for the Kondo droplets shown in the insets with lattice spacings **a** $\Delta {r}_{1}=4{a}_{0}\approx 10.2$ Å (droplet 1), and **b** $\Delta {r}_{2}=4\sqrt{3}{a}_{0}\approx 17.7$ Å (droplet 2) built on the Cu(111) close-packed surface with an exact 6-fold symmetry. Topographic data in insets uses same colorbar as Fig. 1. **c**, **d** Theoretical ${\mathrm{d}}I/{\mathrm{d}}V$ lineshapes for the Kondo droplets shown in the insets of **a**, **b**, respectively. Note that the experimental data show a weak scattering peak centered at ${V}_{0}=-10$ mV for droplet 1, and ${V}_{0}=-8$ mV for droplet 2, which arises from the presence of proximal step edges, and is not directly related to the Kondo resonance. A detailed discussion of how the theoretical lineshapes are obtained is provided in Supplementary Note 4.

While our theoretical results predict the largest difference in the strength of the Kondo screening between droplets with $\Delta r=4\sqrt{3}{a}_{0}$ and $\Delta r=3{a}_{0}$, we find experimentally that Co lattices with $n \, < \, 4$ are insufficiently stable for detailed spectroscopy. On the other hand, for the two lattice types shown in Fig. 2a, b, the $n=4$ family yields nearest-neighbor magnetic moment distances closest to and straddling ${\lambda }_{F}/2 \sim 15$ Å, corresponding to the screening limit of $\sim \!\!1$ itinerant electron per spin impurity. Moreover, our theoretical results predict a large difference in correlated behavior between the two types of $n=4$ droplets (Fig. 3a), contrasting a nearly uncorrelated droplet (dashed vertical black arrow) with a maximally coherent state (solid vertical black arrow). Accordingly, we use atomic manipulation techniques to build two Kondo droplets, each consisting of three rings of Co adatoms with adatom distances of $\Delta {r}_{{\rm{1}}}=4{a}_{0}\approx 10.2$ Å (droplet 1) and $\Delta {r}_{{\rm{2}}}=4\sqrt{3}{a}_{0}\approx 17.7$ Å (droplet 2), as shown in the insets of Fig. 4a, b, respectively, where ${a}_{0}=2.55$ Å is the surface nearest-neighbor spacing for Cu(111) at 4.2 K. In Fig. 4a, b, we present the experimental ${\mathrm{d}}I/{\mathrm{d}}V$ lineshapes measured via STS at the center and edge sites of these droplets, and for comparison, that of an isolated Co adatom. The ${\mathrm{d}}I/{\mathrm{d}}V$ lineshapes for these two droplets exhibit striking differences: the widths of the Kondo resonances in droplet 2 are significantly enhanced over that of the isolated Co adatom, with the center showing a larger $\Delta {E}_{{\rm{K}}}$ than the edge site. Using a Fano fit^28^ to extract the width of the Kondo resonance (Supplementary Note 2), and setting $\Delta {E}_{{\rm{K}}}={k}_{\rm{B}}{T}_{{\rm{K}}}$ (ref. ^1^), we find that the largest Kondo temperature ${T}_{{\rm{K}}}=72\pm 6$ K is observed at the droplet center, representing an $\sim {\!\!} 35$% enhancement over the ${T}_{{\rm{K}}}=53\pm 5$ K of an isolated Co adatom. In stark contrast to this behavior, the corresponding ${\mathrm{d}}I/{\mathrm{d}}V$ lineshapes in droplet 1 are nearly identical to that of an isolated Co adatom, and the widths of their respective Kondo resonances are the same within experimental error. In Fig. 4c, d, we present the theoretically computed ${\mathrm{d}}I/{\mathrm{d}}V$ lineshapes^24,25,30,31^ (see Supplementary Note 4), which well reproduce the experimental results shown in Fig. 4a, b. These results confirm the theoretically predicted enhancement of the width of the Kondo resonance for $\Delta {r}_{{\rm{2}}}=4\sqrt{3}{a}_{0}$ (see Fig. 3a).

### Kondo echo as a signature of quantum coherence

**Fig. 5:**
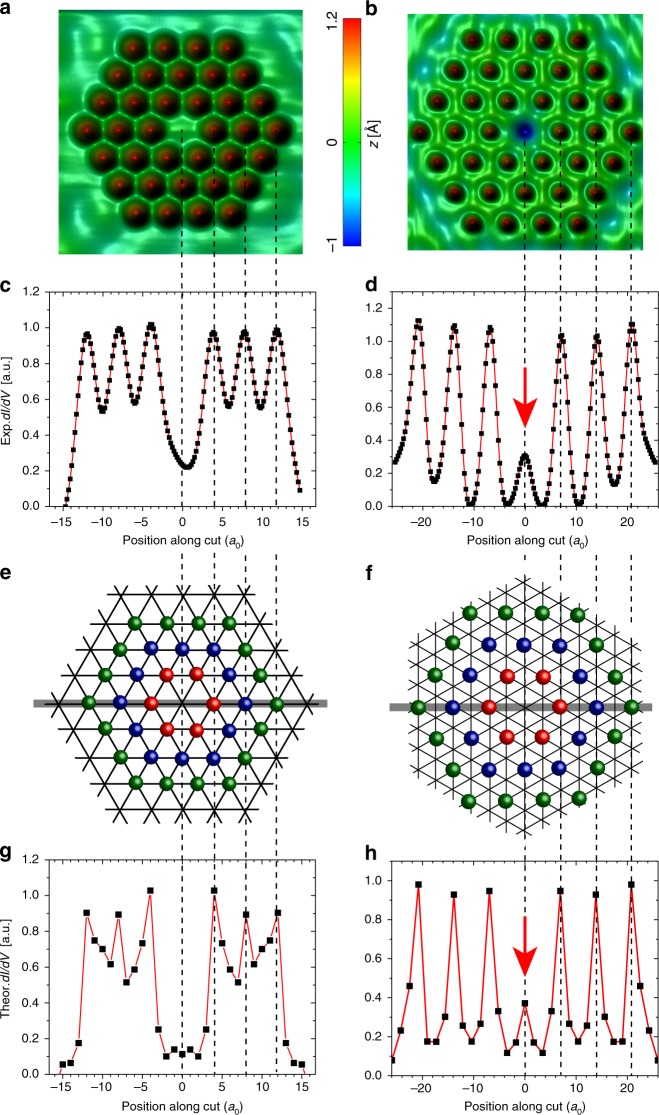
Emergence of a Kondo echo. Experimental Kondo droplets for **a** $\Delta {r}_{{\rm{1}}}=4{a}_{0}$ (droplet 1), and **b** $\Delta {r}_{{\rm{2}}}=4\sqrt{3}{a}_{0}$ (droplet 2) with a Kondo hole (vacancy) at the center site. **c**, **d** Experimental ${\mathrm{d}}I/{\mathrm{d}}V$ at $V=10$ mV acquired along a horizontal cut through the Kondo droplets in **a**, **b**, respectively. Theoretical Kondo droplets for **e** $\Delta {r}_{{\rm{1}}}=4{a}_{0}$, and **f** $\Delta {r}_{{\rm{2}}}=4\sqrt{3}{a}_{0}$ with a Kondo hole (vacancy) at the center site. **g**, **h** Theoretical ${\mathrm{d}}I/{\mathrm{d}}V$ at $E=10$ meV along a horizontal cut (gray line) through the Kondo droplets in **e**, **f**, respectively. The spatial ($x$-axis) scale is the same in panels **a**, **c**, **e**, **g** (**b**, **d**, **f**, **h**). A detailed discussion of how the theoretical ${\mathrm{d}}I/{\mathrm{d}}V$ was computed is provided in Supplementary Note 5.

The creation of a coherent Kondo droplet for $\Delta {r}_{{\rm{2}}}=4\sqrt{3}{a}_{0}$ (droplet 2) leads to another unique effect—the formation of a Kondo echo. When a magnetic adatom is removed from the droplet’s center to create a vacancy, or a Kondo hole (as shown in Fig. 5a, b), the spectral signature of the Kondo effect can be observed nonlocally at this site as a Kondo echo. To demonstrate this, we present in Fig. 5c, d the experimentally measured differential conductance, ${\mathrm{d}}I/{\mathrm{d}}V$, along a line through the center of the uncorrelated Kondo droplet 1 with $\Delta {r}_{{\rm{1}}}=4{a}_{0}$ and the coherently coupled Kondo droplet 2 with $\Delta {r}_{{\rm{2}}}=4\sqrt{3}{a}_{0}$ (see horizontal gray lines in Fig. 5e, f), respectively, at $E=10$ meV, corresponding approximately to the maximal ${\rm{d}}I/{\rm{d}}V$ of the Kondo resonance (see dashed line in Fig. 3b). A comparison of Fig. 5b, d, which both have the same spatial ($x$-axis) scale, shows that ${\mathrm{d}}I/{\mathrm{d}}V$ exhibits a peak at the sites of each magnetic adatom. Interestingly enough, however, an additional peak in ${\mathrm{d}}I/{\mathrm{d}}V$ – the Kondo echo – occurs at the Kondo hole site in the center of droplet 2. This Kondo signal represents the correlated signature of the Kondo screening cloud of electrons that is macroscopically coherent over the entire droplet. As further evidence of this effect, we show that Kondo droplet 1 (see Fig. 5a), in which the Kondo resonances are essentially decoupled from each other, exhibits no Kondo signal at the center vacancy (see Fig. 5c). Our theoretical results for ${\mathrm{d}}I/{\mathrm{d}}V$ along the same line for the same Kondo droplets with a Kondo hole at the center (see Fig. 5e, f and Supplementary Note 5 for details) very well reproduce these experimental findings (see Fig. 5g, h), and in particular, confirm the existence of a Kondo echo at the vacancy site of Kondo droplet 2 (Fig. 5h), and its absence in droplet 1 (Fig. 5g). The existence or absence of a Kondo echo in droplets 2 and 1, respectively, is further confirmed by the experimentally measured ${\mathrm{d}}I/{\mathrm{d}}V$ lineshape at the center vacancy sites shown in Supplementary Fig. 5 of Supplementary Note 6. While ${\mathrm{d}}I/{\mathrm{d}}V$ at the vacancy site of droplet 2 still exhibits a characteristic Kondo resonance, and hence a Kondo echo, ${\mathrm{d}}I/{\mathrm{d}}V$ at the vacancy site of droplet 1 does not. These results thus demonstrate that the existence of a nonlocal Kondo echo reflects the presence of an extended Kondo cloud that is pervasive and coherent over the entire droplet.

## Discussion

The ability to manipulate the strength of Kondo screening, and hence the Kondo temperature, by varying the size, shape, or lattice constant of Kondo droplets opens a venue for the exploration and quantum engineering of many-body effects, and the many ensuing unconventional properties and phases exhibited by strongly correlated electron materials. Future studies will focus on the possibility to tune the competition between Kondo screening and antiferromagnetic correlations – which is at the heart of the heavy-fermion problem^8^ – in the same manner as Kondo screening by itself can be manipulated. The creation of Kondo droplets that balance these two competing phenomena such that the addition of a single magnetic adatom could tip the balance one way or another, presents an exciting opportunity to explore this competition. Furthermore, Kondo screening enables the emergence of unconventional superconductivity in many heavy-fermion materials, raising the possibility to create superconducting correlations in Kondo droplets, and manipulate the incipient spatial structure of the superconducting order parameter. The recent development of Josephson scanning tunneling spectroscopy^32,33^ would be a suitable tool to probe the emergence of such superconducting correlations on the atomic scale, opening paths for exploring and manipulating not only the emergence of Kondo lattices and heavy-fermion physics, but also that of unconventional superconducting phases.

## Methods

### Theoretical methods

The theoretical results for the local hybridization and Greens functions were obtained using a real-space large-$N$ expansion of the Kondo Hamiltonian (for details see Supplementary Note 1). The differential conductance, ${\mathrm{d}}I/{\mathrm{d}}V$, was computed assuming simultaneous tunneling into the conduction electron and magnetic $d$-orbitals, with tunneling amplitudes ${t}_{c}$ and ${t}_{f}$, respectively.

### Experimental methods and procedures

The single crystal Cu(111) surface was prepared by repeated cycles of Ar+ ion sputtering and annealing in ultrahigh vacuum. After cooling the sample to 4.2 K, an electron beam evaporator was used to dose Co to $\sim {\!} 4$ atoms/(100 Å)^2^ coverage for the Kondo droplet experiments. The STS tip was then used as an atom manipulator to construct nanoscopic Co lattices comprises 36 Co atoms each of varying geometry. Using scanning tunneling spectroscopy, the differential conductance, ${\mathrm{d}}I/{\mathrm{d}}V$ was measured. Typical spectroscopic measurement parameters: ${R}_{{\rm{T}}}=100$ MΩ –1 GΩ at $V=10$ mV, ${V}_{\text{ac}}=250\,$µV – 1 mV rms, $f{\!}={\!}200\!-\!\!1000$ Hz. ${\mathrm{d}}I/{\mathrm{d}}V$ image maps and linecuts were acquired simultaneously with associated topographs by applying an a.c. modulation (typically 250 µV to 1 mV r.m.s. at 201 or 1007 Hz) at a frequency exceeding the bandwidth of the feedback loop, and recording the lock-in detected ${\mathrm{d}}I/{\mathrm{d}}V$ (conductance map) along with tip height (topograph) at fixed d.c. bias $V=10$ mV while the tip was scanned in closed-loop constant-$I$ mode. Such scans provided a Kondo signal map proportional to the depletion of the local density of states at ${E}_{\rm{ F}}$ due to the Kondo resonance.

## Supplementary information

Supplementary Information

## Data Availability

The data that support the findings of this study are available from the authors on reasonable request; see author contributions for specific data sets.

## Code availability

The codes that were employed in this study are available from the authors on reasonable request.
