# Supplementary material for: Quantum engineered Kondo lattices
Source: Nat Commun. 2019 Dec 6;10:5588. doi: 10.1038/s41467-019-13446-1 (PMC6898616; doi:10.1038/s41467-019-13446-1)
Supplement: Supplementary file 1 — Supplementary Information [file 41467_2019_13446_MOESM1_ESM.pdf]

Quantum Engineered Kondo Lattices

Figgins et al.

Quantum Engineered Kondo Lattices

Supplemental Information

Jeremy Figgins[1], Laila S. Mattos[2,3], Warren Mar[2,4], Yi-Ting Chen[2,5], Hari C. Manoharan[2,3], and Dirk K. Morr[1]

[1] *Department of Physics, University of Illinois at Chicago, Chicago, Illinois 60607, USA*
[2] *Stanford Institute for Materials and Energy Sciences, SLAC National Accelerator Laboratory, Menlo Park, California 94025, USA*
[3] *Department of Physics, Stanford University, Stanford, California 94305, USA*
[4] *Department of Electrical Engineering, Stanford University, Stanford, California 94305, USA and*
[5] *Department of Applied Physics, Stanford University, Stanford, California 94305, USA*

Supplementary Note 1: Theoretical Formalism

We consider Co adatoms placed on a metallic Cu(111) surface in the form of highly ordered, hexagonal Kondo droplets (see Figs. 1 and 2 of the main text). Kondo screening arises from the coupling of the magnetic adatoms to the two-dimensional (2D) surface band. Such a systems is described by the Kondo Hamiltonian[1–8]

$$\mathcal{H} = \sum_{\mathbf{r},\mathbf{r}',\sigma} \left(-t_{\mathbf{r}\mathbf{r}'} - \mu\delta_{\mathbf{r}\mathbf{r}'}\right) c^\dagger_{\mathbf{r},\sigma} c_{\mathbf{r}',\sigma} + J{\sum_{\mathbf{r}}}' \mathbf{S}^{\mathrm{K}}_{\mathbf{r}} \cdot \mathbf{s}^{\mathrm{c}}_{\mathbf{r}} \ , \tag{1}$$

where $c^\dagger_{\mathbf{r},\sigma}(c_{\mathbf{r},\sigma})$ creates (annihilates) a conduction electron with spin σ at site $\mathbf{r}$ on the Cu(111) surface. Here, $t_{\mathbf{r}\mathbf{r}'} = 0.924$ eV is the fermionic hopping amplitude between nearest-neighbor sites on the triangular Cu (111) surface lattice, and $\mu = -5.13$ eV is its chemical potential[9], yielding a Fermi wavelength of $\lambda_{\mathrm{F}} \approx 11.5a_0$, where a_0 is the Cu lattice constant. Moreover, $J > 0$ is the Kondo coupling, and $\mathbf{S}^{\mathrm{K}}_{\mathbf{r}}$ and $\mathbf{s}^{\mathrm{c}}_{\mathbf{r}}$ are the spin operators of the magnetic (Kondo) adatom and the conduction electron at site $\mathbf{r}$, respectively. The primed sum runs over the locations of the magnetic adatoms only. Finally, to reproduce the line shape and width of the Kondo resonance measured experimentally[10] for a single Co adatom on a Cu(111) surface, we employ $J = 3.82$ eV and $N = 4$ (see Fig. 4 of the main text and Ref. 11 for a more detailed discussion).

We note that the Ruderman - Kittel - Kasuya - Yosida (RKKY) interaction between magnetic moments[2,4,7] for the 2D Cu(111) surface band decays rapidly with increasing distance[12], and is significantly smaller than $k_B T_{\mathrm{K}}$ for the relevant inter-adatom distances considered in the main text, in agreement with experimental results[13]. In particular, already for inter-adatom distances of two lattice constants, the RKKY interaction $I_{\mathrm{RKKY}}(2a_0) \approx 2.3$ meV is smaller than $k_B T_{\mathrm{K}} \approx 4.7$ meV for a single Co adatom on a Cu(111) surface[14]. For the inter-adatom distances considered in Figs. 4 and 5 of the main text, I_{RKKY} is less than 2% of $k_B T_{\mathrm{K}}$[14]. For these reasons, the effect of the RKKY interaction on the hybridization and the dI/dV lineshape is negligible, and therefore can be omitted in our calculations.

Starting from the Hamiltonian in Eq.(1), a systematic large-N expansion[3,4,6,8,15–18] can be achieved by generalizing the spin operators to $SU(N)$ and representing them using Abrikosov pseudofermion operators

$$\mathbf{S}^{\mathrm{K}}_{\mathbf{r}} = \sum_{\alpha,\beta} f^\dagger_{\mathbf{r},\alpha} \boldsymbol{\sigma}_{\alpha,\beta} f_{\mathbf{r},\beta} \quad \mathbf{s}^{\mathrm{c}}_{\mathbf{r}} = \sum_{\alpha,\beta} c^\dagger_{\mathbf{r},\alpha} \boldsymbol{\sigma}_{\alpha,\beta} c_{\mathbf{r},\beta} \ , \tag{2}$$

where $\alpha, \beta = 1, ..., N$ and $\boldsymbol{\sigma}_{\alpha,\beta}$ is a vector whose $(N^2 - 1)$ elements are the generators of $SU(N)$ in the fundamental representation. Each generator is represented by an $(N \times N)$ matrix with indices α, β. Here, $f^\dagger_{\mathbf{r},\alpha}$ $(f_{\mathbf{r},\alpha})$ creates (annihilates) a pseudofermion in the magnetic d-orbitals of the Co adatom characterized by the spin quantum number α. To ensure the existence a magnetic moment, one needs to satisfy the constraint that each adatom site is singly-occupied, i.e.,

$$\hat{n}_{\mathrm{f}}(\mathbf{r}) = \sum_{\alpha} f^\dagger_{\mathbf{r},\alpha} f_{\mathbf{r},\alpha} = 1 \ . \tag{3}$$

Inserting the representations of Eq.(2) into the Hamiltonian, Eq.(1), yields quartic fermionic interaction terms. On the mean-field level, we decouple these terms by introducing the expectation value

$$s(\mathbf{r}) = \frac{J}{2} \sum_{\alpha} \langle f^\dagger_{\mathbf{r},\alpha} c_{\mathbf{r},\alpha} \rangle \ , \tag{4}$$

where $s(\mathbf{r})$ describes the local hybridization between the conduction electron states and the magnetic f-electron states. $s(\mathbf{r})$ is a measure of the strength of the Kondo screening, with $s(\mathbf{r}) = 0$ representing an unscreened magnetic moment at site $\mathbf{r}$. The constraint in Eq.(3) is enforced on the mean-field level, yielding $n_{\rm f}(\mathbf{r}) = \langle \hat{n}_{\rm f}(\mathbf{r})\rangle = 1$, by adding a Largrange multiplier in the form of the term $\sum_{\mathbf{r},\alpha} \varepsilon_{\rm f}(\mathbf{r}) f^\dagger_{\mathbf{r},\alpha} f_{\mathbf{r},\alpha}$ to the Hamiltonian in Eq.(1), where $\varepsilon_{\rm f}(\mathbf{r})$ represents the on-site energy of the f-electron states. The resulting Hamiltonian is quadratic and can therefore be diagonalized in real space. However, since the lifetime of the conduction and magnetic d-orbital states plays a major role in determining the dI/dV lineshape, we account for them by rewriting the above equation for $s(\mathbf{r})$ [Eq.(4)] and the constraint $n_{\rm f}(\mathbf{r}) = 1$ in the form

$$s(\mathbf{r}) = -\frac{J}{\pi} \int_{-\infty}^{\infty} d\omega \; n_{\rm F}(\omega) \; {\rm Im} G_{\rm cf}(\mathbf{r},\mathbf{r},\omega) \; ;$$
$$n_{\rm f}(\mathbf{r}) = -\frac{1}{\pi} \int_{-\infty}^{\infty} d\omega \; n_{\rm F}(\omega) \; {\rm Im} G_{\rm ff}(\mathbf{r},\mathbf{r},\omega) = 1 \; , \tag{5}$$

where $n_{\rm F}(\omega)$ is the Fermi distribution function, and

$$\hat{G}_{\rm ff}(\omega) = \left[\hat{g}_{\rm ff}^{-1}(\omega) - \hat{s}\hat{g}_{\rm cc}(\omega)\hat{s}\right]^{-1} \; ;$$
$$\hat{G}_{\rm cc}(\omega) = \left[\hat{g}_{\rm cc}^{-1}(\omega) - \hat{s}\hat{g}_{\rm ff}(\omega)\hat{s}\right]^{-1} \; ;$$
$$\hat{G}_{\rm cf}(\omega) = -\hat{g}_{\rm cc}(\omega)\hat{s}\hat{G}_{\rm ff}(\omega) \; . \tag{6}$$

Here, $\hat{G}_{xy}(\omega)$ $(x, y = {\rm c}, {\rm f})$ are the retarded Greens function matrices in real space with $G_{xy}(\mathbf{r},\mathbf{r},\omega)$ being the (r, r) element of the matrix $\hat{G}_{xy}$. $\hat{g}_{\rm ff}$ and $\hat{g}_{\rm cc}$ are the unhybridized Greens function matrices, and the spin degeneracy has been accounted for by dropping the spin index in the matrices. The hybridization matrix $\hat{s}$ is only non-zero at the sites where a magnetic adatom is located. A finite lifetime, $\tau_{\rm c,f}$, of the conduction and d-orbital states is then introduced in the above Greens functions via the scattering rate $\Gamma_{\rm c,f} = \hbar/\tau_{\rm c,f}$ yielding

$$\hat{g}_{\rm ff}(\mathbf{r},\mathbf{r},\omega) = \left[\omega - \varepsilon_{\rm f}(\mathbf{r}) + i\Gamma_{\rm f}\right]^{-1} \tag{7}$$
$$\hat{g}_{\rm cc}(\mathbf{k},\omega) = \left[\omega - \varepsilon_{\mathbf{k}} + i\Gamma_{\rm c}\right]^{-1} \; . \tag{8}$$

with $\hat{g}_{\rm cc}(\mathbf{r},\mathbf{r},\omega)$ being obtained from $\hat{g}_{\rm cc}(\mathbf{k},\omega)$ via Fourier transform. To reproduce the experimentally measured dI/dV lineshapes shown in Fig. 4 of the main text, we used $\Gamma_{\rm f} = 4$ meV and $\Gamma_{\rm c} = 65$ meV. Eqs.(5) - (8) are a closed set of equations that can now be self-consistently solved to obtain the local hybridizations, $s(\mathbf{r})$ and f-electron energies $\varepsilon_{\rm f}(\mathbf{r})$. We note that for a single Kondo impurity, this formalism is identical to the saddle-point approximation of the path-integral approach of Ref.[15] in the large-N approximation which becomes exact in the limit $N \to \infty$.

Supplementary Note 2: Differential Conductance dI/dV, the Fano formula, and the width of the Kondo resonance

To compute the differential conductance, dI/dV[11,19–21], measured in scanning tunneling spectroscopy (STS) experiments on Kondo systems[22] and heavy fermion materials[23–25], we define the spinor $\Psi^\dagger_{\mathbf{k},\alpha} = (c^\dagger_{\mathbf{k},\alpha}, f^\dagger_{\mathbf{k},\alpha})$ and the Green's function matrix $\hat{G}_\alpha(\mathbf{k},\tau) = -\langle T_\tau \Psi_{\mathbf{k},\alpha}(\tau)\Psi^\dagger_{\mathbf{k},\alpha}(0)\rangle$. With $t_{\rm c}$ and $t_{\rm f}$ being the amplitudes for electronic tunneling from the scanning tunneling microscope (STM) tip into the conduction electron bands of the Cu(111) surface and the magnetic d-orbitals of the Co adatom at site $\mathbf{r}$, respectively, one obtains[11]

$$\frac{dI(\mathbf{r},\omega)}{dV} = -\frac{e^2}{\hbar} N_{\rm t} \sum_\alpha \sum_{i,j=1}^{2} \left[\hat{t}\, {\rm Im}\hat{G}_\alpha(\mathbf{r},\mathbf{r},\omega)\, \hat{t}\right]_{ij} \tag{9}$$

where $\hat{t} = \begin{pmatrix} t_{\rm c} & 0 \\ 0 & t_{\rm f} \end{pmatrix}$, $\hat{G}_\alpha(\mathbf{r},\mathbf{r},\omega)$ is the local, retarded Greens function matrix, and $N_{\rm t}$ is the STM tip's density of states, which is taken to be constant. In the weak-tunneling limit, $t_{\rm c}, t_{\rm f} \to 0$, we obtain

$$\frac{dI(\mathbf{r},V)}{dV} = \frac{2\pi e^2}{\hbar} N_{\rm t} \left[t_{\rm c}^2 N_{\rm c}(\mathbf{r},eV) + t_{\rm f}^2 N_{\rm f}(\mathbf{r},eV) + 2t_{\rm c}t_{\rm f} N_{\rm cf}(\mathbf{r},eV)\right] \tag{10}$$

where

$$N_{\rm c}(\mathbf{r},\omega) = -\frac{1}{\pi}{\rm Im}G_{\rm cc}(\mathbf{r},\mathbf{r},\omega)$$
$$N_{\rm f}(\mathbf{r},\omega) = -\frac{1}{\pi}{\rm Im}G_{\rm ff}(\mathbf{r},\mathbf{r},\omega)$$
$$N_{\rm cf}(\mathbf{r},\omega) = -\frac{1}{\pi}{\rm Im}G_{\rm cf}(\mathbf{r},\mathbf{r},\omega) \qquad (11)$$

with $N_{\rm c}$ and $N_{\rm f}$ being the density of states of the conduction and f-electron (d-orbital) states, respectively. To reproduce the line shape and width of the Kondo resonance measured experimentally[10] for a single Co adatom on a Cu(111) surface, we employ $J = 3.82$ eV and $N = 4$ [see Fig. 4 of the main text and Refs. 11 and 21].

To extract the width, $\Delta E_{\rm K}$ of the Kondo resonances (shown in Figs. **2e** and **3a** of the main text), we have fitted the theoretical dI/dV lineshapes obtained from Eq.(10) (shown, for example, in Fig. 3**b** of the main text) by the Fano formula[26]

$$\frac{dI(\mathbf{r},V)}{dV} = y_0 + cV + B\frac{\left(\frac{eV-\omega_0}{\Delta E_{\rm K}}+q\right)^2}{\left(\frac{eV-\omega_0}{\Delta E_{\rm K}}\right)^2+1} \qquad (12)$$

where $\Delta E_{\rm K}$ measures the width of the Kondo resonance and q its asymmetry. Here, we have added the first two terms on the r.h.s. of Eq.(12) to the Fano formula to account for an off-set and a sloping background, respectively. The Fano formula yields an acceptable fit to the theoretically computed dI/dV, as shown in Supplementary Fig. 1 for the center sites of three droplets with $\Delta r = 3a_0$, $\Delta r = 4a_0$, and $\Delta r = 4\sqrt{3}a_0$. $\Delta E_{\rm K}$ extracted from these fits is displayed in Fig. 3**a** of the main text. We find that changing the tunneling ratio, $t_{\rm f}/t_{\rm c}$, changes the values for ω_0 and q, but has only a weak effect on the width of the resonance $\Delta E_{\rm K}$.

The slight discrepancy between the Fano formula fit and the actual theoretically computed differential conductance can be attributed to the fact that the derivation of the Fano formula is only valid (a) in the limit of a single magnetic adatom and (b) if both the real and imaginary parts of the conduction electron Greens function are energy independent in the vicinity of the Kondo resonance. Indeed in this limit, we find that the theoretical form for the

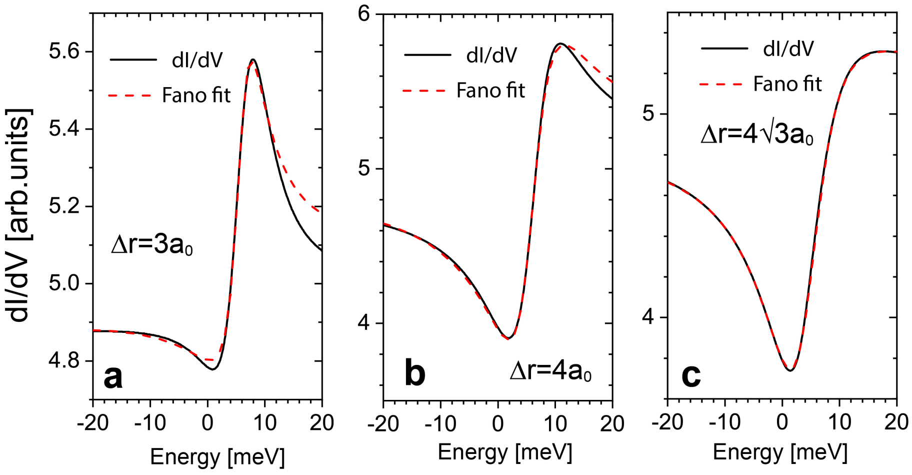

Supplementary Figure 1. Theoretical dI/dV (black line) from Eq.(10) and Fano fit (red dashed line) from Eq.(12) at the center of a droplet with 3 rings and **a** $\Delta r = 3a_0$, **b** $\Delta r = 4a_0$, and **c** $\Delta r = 4\sqrt{3}a_0$. $\Delta E_{\rm K}$ extracted from these fits is displayed in Fig. 3**a** of the main text, with the vertical error bars indicating the uncertainty in extracting $\Delta E_{\rm K}$.

differential conductance in Eq.(10) can be written in the same form as the Fano formula, Eq.(12), and is given by

$$\frac{dI(\mathbf{r},V)}{dV} = \frac{2\pi e^2}{\hbar}N_{\rm t}t_{\rm c}^2N_0\frac{\left(\frac{eV-\omega_0}{\Delta E_{\rm K}}+q\right)^2}{\left(\frac{eV-\omega_0}{\Delta E_{\rm K}}\right)^2+1} \qquad (13)$$

where

$$\Delta E_{\rm K} = s^2 \pi N_0 \qquad q = -\frac{s^2 {\rm Re} g_{\rm cc}(\mathbf{R}, \omega_0) - s\frac{t_{\rm f}}{t_{\rm c}}}{s^2 {\rm Im} g_{\rm cc}(\mathbf{R}, \omega_0)} \ . \tag{14}$$

Here, N_0 is the density of states of the unperturbed conduction band, and $g_{\rm cc}(\mathbf{R}, \omega_0)$ is the retarded Greens function of the conduction band at the site $\mathbf{R}$ of the single magnetic adatom and at energy ω_0. Note that the above formula also shows that the asymmetry of the Kondo resonance, as described by q, can be changed through interference processes between electron tunneling into the magnetic levels, described by $t_{\rm f}$, and the conduction band, described by $t_{\rm c}$. The presence of multiple Kondo screened adatoms in the Kondo droplets, as well as the energy dependence of $g_{\rm cc}(\mathbf{R}, \omega)$ will lead to deviations of the actual dI/dV from the above Fano formula in Eq.(12).

Supplementary Note 3: Quantum Interference and Kondo screening

To demonstrate how quantum interference of the Kondo screening clouds associated with different magnetic adatoms can give rise to variations in the strength of the Kondo screening, as evidenced by the magnitude of the hybridization, s, or the width of the Kondo resonance, $\Delta E_{\rm K}$ (as shown in Figs. 2 and 3 of the main text) we begin by considering

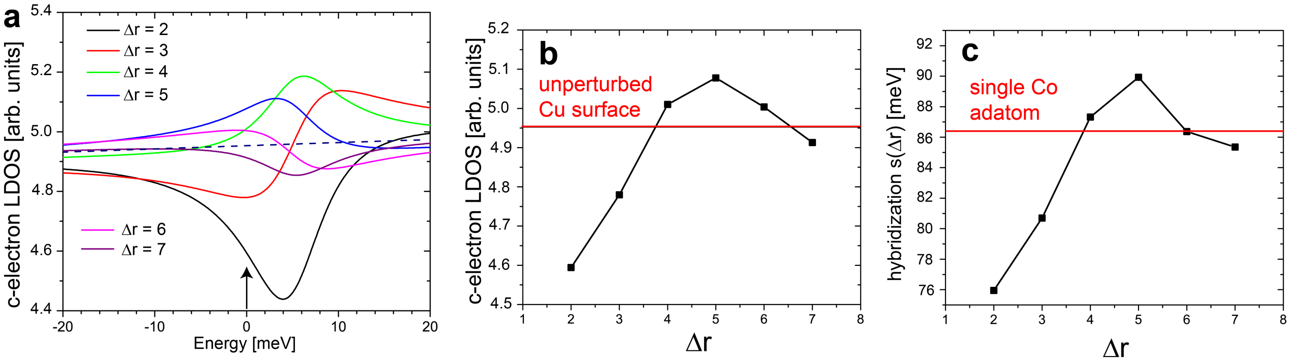

Supplementary Figure 2. **a** Theoretical LDOS of the conduction c-electrons at several distances Δr from a single Co atom on a Cu(111) surface (same parameters as in the main text). **b** c-electron LDOS at $E = 0$ as a function of Δr. The red line represents the LDOS of the unperturbed Cu(111) surface. **c** Theoretically computed hybridization of 2 Co atoms as a function of inter-adatom distance, Δr. The red line shows the hybridization for a single Co adatom.

the effects of a single, Kondo-screened Co adatom on the electronic structure in its vicinity. To this end, we present in Supplementary Fig. 2**a** the local density of states (LDOS) of the conduction c-electrons for several distances Δr from a single, Kondo screened Co adatom located an a Cu(111) surface. The non-monotonic dependence of the LDOS on Δr arises from $2k_{\rm F}r$ scattering of the screening conduction electrons. We next consider the effects on the hybridization when a second Co adatom is added to the system by noting that the magnitude of the hybridization is in general determined by the value of the conduction electron LDOS – the screening band – near the Fermi energy. This LDOS at $E = 0$ as a function of Δr is shown in Supplementary Fig. 2**b**. When a second Co adatom is now placed at a distance Δr from the first impurity where the LDOS is smaller (larger) than in the unperturbed metal (without a Kondo impurity), one expects that the resulting hybridization (which by symmetry is the same for both Co adatoms) is also smaller (larger) than that of a single Co adatom, which is placed on the unperturbed Cu surface. This expectation is confirmed by the explicit calculation of the hybridization of a 2 Co adatom system, as a function of inter-adatom distance, Δr, shown in Supplementary Fig. 2**c**, which shows the same non-monotonic dependence on Δr as the LDOS in Supplementary Fig. 2**b**. In Kondo droplets consisting of multiple Co adatoms, it is the same effect as discussed above for the 2 Co adatom system that leads to the non-monotonic dependence of the hybridization on Δr: it is the quantum interference of the Kondo screening clouds associated with each of the Co adatoms that determines the resulting spatial distribution of the hybridization.

Supplementary Note 4: Theoretical Fits to the Experimentally measured dI/dV

The theoretical fits to the experimental dI/dV results in Figs. 4 and 5 of the main text were obtained with the same set of parameters, $J, \Gamma_{\rm c}$, and $\Gamma_{\rm f}$, as the results shown in Figs. 2 and 3 of the main text. To obtain the theoretical

fits shown in Figs. 4 and 5 of the main text, we note that the experimental data in Fig. 4**a** (Fig. 4**b**) of the main text show a weak scattering peak around $V_0 = -10$ mV ($V_0 = -8$ mV), which arises from the presence of proximal step edges, and is not directly related to the Kondo resonance. The data also exhibit a sloping background which likely

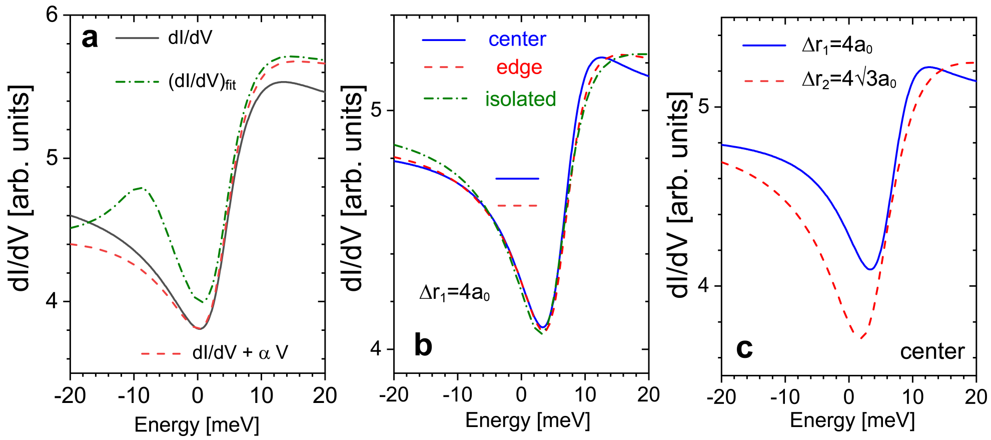

Supplementary Figure 3. **a** Differential conductance at the center site of Kondo droplet 2 with $\Delta r_2 = 4\sqrt{3}a_0$: dI/dV (black line), $dI/dV + \alpha V$ (red dashed line), and $(dI/dV)_{\text{fit}}$ (green dotted-dashed line). **b** dI/dV at the center (blue line) and edge (red dashed line) of Kondo droplet 1 with $\Delta r_1 = 4a_0$, together with dI/dV of an isolated Co adatom (green dotted-dashed line). The widths of the Kondo resonance are approximately equal. Data have been vertically scaled to enable direct comparison of the Kondo resonances' widths. **c** dI/dV at the center of droplet 1 with $\Delta r_1 = 4a_0$ (blue line, $t_f/t_c = 0.025$) and droplet 2 with $\Delta r_2 = 4\sqrt{3}a_0$ (red dashed line, $t_f/t_c = 0.029$), showing a significant difference in the width of the Kondo resonances. The different values for t_f/t_c for droplets 1 and 2 were chosen in order to obtain the same maximum dI/dV.

arises from the bulk rather than the surface bands. Both of these effects were added to the theoretical computed dI/dV obtained from Eq.(10) to obtain the theoretical fit denoted by $(dI/dV)_{\text{fit}}$ shown in Figs. 4 and 5 of the main text and given by

$$\left(\frac{dI}{dV}\right)_{\text{fit}} = \frac{dI}{dV} + \alpha V + \beta L(V) \tag{15}$$

where

$$L(V) = \frac{\Gamma}{(V - V_0)^2 + \Gamma^2} \tag{16}$$

is the Lorentzian peak, and α and β are constants. Experimentally, we find that for a single Co adatom, as well as for droplet 1, the peak is centered at $V_0 = -10$ mV with half-width $\Gamma = 12.5$ mV, while for droplet 2, the peak is centered at $V_0 = -8$ mV with half-width $\Gamma = 5.75$ mV. In Supplementary Fig. 3**a** we show the effects of the sloping background and the spectral peak on the dI/dV lineshape at the center site of lattice 2 (with $\Delta r_2 = 4\sqrt{3}a_0$) by presenting the theoretical dI/dV obtained from Eq.(10) (black line), $dI/dV + \alpha V$ (red dashed line), and $(dI/dV)_{\text{fit}}$ (green dotted-dashed line). Moreover, in Supplementary Fig. 3**b**, we present the theoretical dI/dV for the center and edge sites of Kondo droplet 1 with $\Delta r_1 = 4a_0$ together with that of an isolated Co adatom. A comparison of these dI/dV with $(dI/dV)_{\text{fit}}$ at these three sites shown in Fig. 4**c** of the main text demonstrates that the close similarity of all three lineshapes is not an artefact of the spectral peak. Similarly, a comparison of the theoretical dI/dV at the center sites of Kondo droplets 1 and 2 shown in Supplementary Fig. 3**c** demonstrates that the significant difference in the width of the Kondo resonances between these two droplets (as shown in Figs. 4**c** and **d** of the main text) is also not an artefact of the spectral feature located at V_0.

Supplementary Note 5: Position dependence of dI/dV in closed loop STS experiments

A. Theoretical dI/dV for closed loop STS experiments

To compare our theoretical calculations for the spatial dependence of the differential conductance to the experimental results, shown in Figs. 5**c**,**d** of the main text, we note that the STS experiments are performed in closed loop

mode. This implies, that at every position, the height of the tip from the sample is adjusted to keep the total current

$$I(\mathbf{r}) = \int_0^{V_s} dV \frac{dI(\mathbf{r},V)}{dV} = I_0 \tag{17}$$

for a given set point voltage, V_S, constant. A change in height, however, implies that the tunneling amplitudes become position dependent, and that therefore Eq.(10) needs to be generalized to

$$\frac{dI(\mathbf{r},V)}{dV} = \frac{2\pi e^2}{\hbar} t_c^2(\mathbf{r}) N_t \left[N_c(\mathbf{r},V) + \left(\frac{t_f(\mathbf{r})}{t_c(\mathbf{r})}\right)^2 N_f(\mathbf{r},V) + 2\frac{t_f(\mathbf{r})}{t_c(\mathbf{r})} N_{cf}(\mathbf{r},V) \right] \tag{18}$$

Inserting Eq.(18) into Eq.(17) then yields

$$t_c^2(\mathbf{r}) = I_0 \left\{ \frac{2\pi e^2}{\hbar} N_t \int_0^{V_s} dV \left[N_c(\mathbf{r},V) + \left(\frac{t_f(\mathbf{r})}{t_c(\mathbf{r})}\right)^2 N_f(\mathbf{r},V) + 2\frac{t_f(\mathbf{r})}{t_c(\mathbf{r})} N_{cf}(\mathbf{r},V) \right] \right\}^{-1} \tag{19}$$

and hence

$$\frac{dI(\mathbf{r},V)}{dV} = I_0 \frac{N_c(\mathbf{r},V) + \left(\frac{t_f(\mathbf{r})}{t_c(\mathbf{r})}\right)^2 N_f(\mathbf{r},V) + 2\frac{t_f(\mathbf{r})}{t_c(\mathbf{r})} N_{cf}(\mathbf{r},V)}{\int_0^{V_s} dV \left[N_c(\mathbf{r},V) + \left(\frac{t_f(\mathbf{r})}{t_c(\mathbf{r})}\right)^2 N_f(\mathbf{r},V) + 2\frac{t_f(\mathbf{r})}{t_c(\mathbf{r})} N_{cf}(\mathbf{r},V) \right]} \tag{20}$$

It is this normalized differential conductance, which is shown in Figs. 5**g,h** of the main text and accounts for the experimental closed loop mode that needs to be compared to the experimentally measured dI/dV linecuts through a Kondo hole droplet, shown in Figs. 5**c,d** of the main text. For our theoretical results, we used $t_f/t_c = 0.03$ at the sites of the Co adatoms, and $t_f = 0$ otherwise.

B. Spatial variations in t_f/t_c

We note that when the height of the tip is varied, both $t_c(\mathbf{r})$ and $t_f(\mathbf{r})$ can in general change, which can lead to variations in t_f/t_c not only between the experimentally studied droplets, but also within the same droplet. To

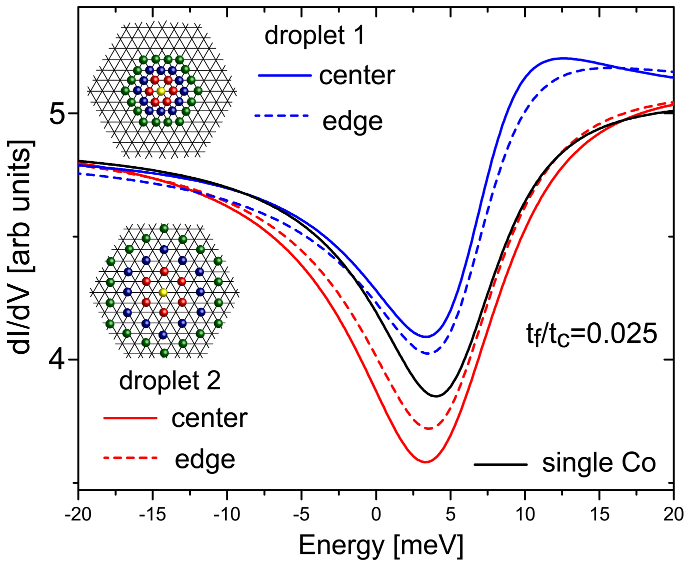

Supplementary Figure 4. Theoretical dI/dV curves with $t_f/t_c = 0.025$ at the center and edge sites for the intact droplet 1 and droplet 2 (shown in the insets), as well as for a single, isolated Co adatom.

demonstrate this, we consider the intact Kondo droplets (i.e., the droplets without a center vacancy) shown in Fig. 4 of the main text. From a comparison of our theoretical results with the experimental dI/dV lineshapes shown in

Figs. 4**a,b** of the main text for the Kondo droplets with $\Delta r_1 = 4a_0$ (droplet 1) and $\Delta r_2 = 4\sqrt{3}a_0$ (droplet 2), respectively, we find that t_f/t_c is slightly larger in droplet 2 than in droplet 1. Specifically, we obtain for droplet 1 $t_f/t_c = 0.025$ both at the center and edge sites, while for droplet 2 we obtain $t_f/t_c = 0.0327$ at the center site, and $t_f/t_c = 0.03$ at the edge site. Finally, for an isolated Co atom, we obtain $t_f/t_c = 0.0275$.

To show that these small changes in t_f/t_c are related to changes in the tip height (due to the closed loop mode in which the STS experiments are performed) between the different spatial locations for which dI/dV lineshapes are shown in Fig. 4 of the main text, we computed the dI/dV lineshapes for all spatial positions with the same value of t_f/t_c, as shown in Supplementary Fig. 4. Given the experimentally used set point voltage of $V_s = 10$ meV, it immediately follows that if t_f/t_c were constant, that then the total current [see Eq.(17)] flowing through the STS tip would be larger for droplet 1 than for an isolated Co atom, and larger for an isolated Co atom than for droplet 2. This implies that in order to measure the same total current in the droplets and for an isolated Co atom, the tip height needs to be the smallest for droplet 2, followed by the isolated Co atom and then droplet 1. Given our analysis above, this suggests that the ratio t_f/t_c systematically increases with decreasing tip height, likely due to the increasing overlap of the tip orbitals with the Co d-orbitals.

Supplementary Note 6: dI/dV at the center site vacancy

In Fig. 5 of the main text, we show that when a magnetic adatom is removed from the droplet's center to create a vacancy, or Kondo hole [see Supplementary Figs. 5**a, b**], the coherent droplet 2 [see Supplementary Fig. 5**b**] with $\Delta r_2 = 4\sqrt{3}a_0$ exhibits a Kondo echo at the center site, while the uncorrelated droplet 1 [see Supplementary Fig. 5**a**] with $\Delta r_1 = 4a_0$ does not. This conclusion is further supported by the experimentally measured dI/dV lineshapes at the center site vacancies for droplets 1 and 2 which are shown in Supplementary Fig. 5**c**. In particular, while the

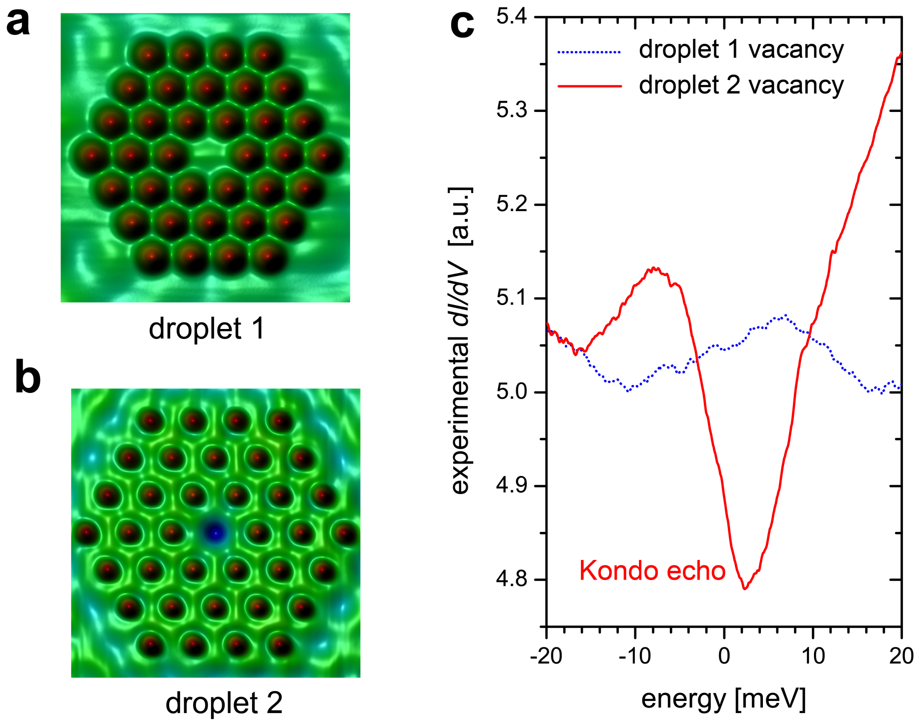

Supplementary Figure 5. Kondo droplets with a center site vacancy (Kondo hole) and **a** $\Delta r_1 = 4a_0$ (droplet 1), and **b** $\Delta r_2 = 4\sqrt{3}a_0$ (droplet 2). **c** dI/dV at the center vacancy sites of droplets 1 and 2.

dI/dV lineshape at the vacancy site of the coherent droplet 2 still exhibits a characteristic Kondo resonance – the Kondo echo – the dI/dV lineshape at the vacancy site of droplet 1 does not. This further supports our conclusion that the observation of a Kondo echo at the center vacancy site is a characteristic signature of a coherent Kondo droplet.

[1] Kondo, J. Resistance Minimum in dilute magnetic alloys, *Prog. Theo. Phys.* **32**, 37 (1964).
[2] Doniach, S. The Kondo lattice and weak antiferromagnetism. *Physica B* **91**, 231 (1977).
[3] Coleman, P. 1/N expansion for the Kondo lattice. *Phys. Rev.* **28**, 5255 (1983).
[4] Hewson, A.C. The Kondo Problem to Heavy Fermions *(Cambridge University Press, Cambridge, England, 1993).*
[5] Si, Q., Rabello, A., Ingersent, K. & Lleweilun Smith J. Local fluctuations in quantum critical metals. *Phys. Rev. B*, **68**, 115103 (2003).
[6] Senthil, T., Vojta, M. & Sachdev, S. Weak magnetism and non-Fermi liquids near heavy-fermion critical points. *Phys. Rev. B* **69**, 035111 (2004).
[7] Ohashi, T., Suga, S. & Kawakami, N. Magnetic properties of a Kondo insulator with RKKY interaction: extended dynamical mean field study. *J. Phys.: Condens. Matter* **17**, 4547 (2005).
[8] Paul, I., Pepin, C. & Norman, M.R. Kondo breakdown and hybridization fluctuations in the Kondo-Heisenberg lattice. *Phys. Rev. Lett.* **98**, 026402 (2007).
[9] Gomes, K.K., Mar, W., Ko, W., Guinea, F. & Manoharan, H.C. Designer Dirac fermions and topological phases in molecular graphene. *Nature* **483**, 306 (2012).
[10] Manoharan, H.C., Lutz, C.P. & Eigler, D.M. Quantum mirages formed by coherent projection of electronic structure. *Nature (London)* **403**, 512 (2000).
[11] Figgins, J. & Morr, D.K. Differential Conductance and Quantum Interference in Kondo Systems *Phys. Rev. Lett.* **104**, 187202 (2010).
[12] Simon, E., Ujfalussy, B., Lazarovits, B., Szilva, A., Szunyogh, L. & Stocks, G. M. Exchange interaction between magnetic adatoms on surfaces of noble metals. *Phys. Rev. B* **83**, 224416 (2011).
[13] Chen, W. , Jamneala, T, Madhavan, V & Crommie, M.F. Disappearance of the Kondo resonance for atomically fabricated cobalt dimers. *Phys. Rev. B* **60**, R8529 (1999).
[14] Simon, E., Ujfalussy, B., Lazarovits, B., Szilva, A., Szunyogh, L. & Stocks, G. M. *private communication, unpublished.*
[15] Read, N. & Newns, D.M. On the solution of the Coqblin-Schrieffer Hamiltonian by the large-N expansion technique, *J. Phys. C* **16**, 3273 (1983).
[16] Bickers, N.E. Review of techniques in the large-N expansion for dilute magnetic alloys. *Rev. Mod. Phys.* **59**, 845 (1987).
[17] Millis, A.J. & Lee, P.A. Large-orbital-degeneracy expansion for the lattice Anderson model. *Phys. Rev. B* **35**, 3394 (1987).
[18] Affleck, I. & Marston, B. Large-n limit of the Heisenberg-Hubbard model: Implications for the high-T_c cuprates. *Phys. Rev. B* **37**, 3774 (1988).
[19] Maltseva, M., Dzero, M. & Coleman, P. Electron Cotunneling into a Kondo Lattice. *Phys. Rev. Lett.* **103**, 206402 (2009).
[20] Wölfle, P., Dubi, Y. & Balatsky, A.V. Tunneling into Clean Heavy Fermion Compounds: Origin of the Fano Line Shape. *Phys. Rev. Lett.* **105**, 246401 (2010).
[21] Morr, D. K. Theory of scanning tunneling spectroscopy: from Kondo impurities to heavy fermion materials *Rep. Prog. Phys.* **80**, 014502 (2017).
[22] Madhavan, V., Chen, W., Jamneala, T., & Crommie, M.F. Local spectroscopy of a Kondo impurity: Co on Au(111), *Phys. Rev. B* **64**, 165412 (2001).
[23] Schmidt, A.R., Hamidian, M.H., Wahl, P., Meier, F., Balatsky, A.V., Garrett, J.D., Williams, T., Luke, G.M. & Davis, J.C. Imaging the Fano lattice to hidden order transition in URu_2Si_2, *Nature* **465**, 570 (2010).
[24] Aynajian, P., da Silva Neto, E. H., Parker, C. V., Huang, Y., Pasupathy, A., Mydosh, J., & Yazdani, A. Visualizing the formation of the Kondo lattice and the hidden order in URu_2Si_2, *PNAS* **107**, 10383 (2010).
[25] Ernst, S., Kirchner, S., Krellner, C., Geibel, C., Zwicknagl, G., Steglich, F. & Wirth, S. Emerging local Kondo screening and spatial coherence in the heavy-fermion metal $YbRh_2Si_2$, *Nature* **474**, 362 (2011).
[26] Fano, U. Effects of Configuration Interaction on Intensities and Phase Shifts. *Phys. Rev.* **124**, 1866 (1961).
